# RETRACTION: The Effects of Platelet-Derived Growth Factor-BB on Bone Marrow Stromal Cell-Mediated Vascularized Bone Regeneration

**DOI:** 10.1155/sci/9754568

**Published:** 2025-11-20

**Authors:** Stem Cells International

RETRACTION: M. Zhang, W. Yu, K. Niibe, W. Zhang, H. Egusa, T. Tang, and X. Jiang, “The Effects of Platelet-Derived Growth Factor-BB on Bone Marrow Stromal Cell-Mediated Vascularized Bone Regeneration,” *Stem Cells International* 2018, no. 1 (2018): 3272098, https://doi.org/10.1155/2018/3272098.

The above article, published online on 31 October 2018 in Wiley Online Library (wileyonlinelibrary.com), has been retracted by agreement between the editorial board and John Wiley & Sons Ltd.

The retraction has been agreed following an investigation of the concerns raised by *Actinopolyspora biskrensis* on PubPeer [1], which identified multiple figure concerns as follows:

• There is unexpected similarity between regions of the Lenti-LacZ and the Lenti-PDGF+PD98059 panels in Figure 2c.
• The Lenti-LacZ and the Lenti-PDGF+PD98059 panels in Figure 4a appear to be identical.
• The ERK bands in day 7 of Figure 2b appear to be identical to the ERK bands in Figure 4c, despite the accompanying bands for p-ERK and β-actin being different.
• The day 1 image shown in Figure 5b appears to be identical to the Day 1 Lenti-PDGF image in Figure 3 of [2].

As a result of the investigation, the data and conclusions of this article are considered unreliable.

The authors disagree to the retraction.
